# *SHORTKIT-ML*: A UNIFIED MULTI-PERSPECTIVE FRAMEWORK FOR DETECTING SHORTCUT LEARNING IN MEDICAL IMAGING EMBEDDINGS

**DOI:** 10.64898/2026.04.29.26352053

**Published:** 2026-04-30

**Authors:** Sebastian Cajas, Aldo Marzullo, Sahil Kapadia, Filipe Santos, Felipe Ocampo Osorio, Qingpeng Kong, Alessandro Quarta, Po-Chih Kuo, Milit Patel, Raul Ignacio Rojas Sillery, Leo Anthony Celi

**Affiliations:** 1 MIT Critical Data, Massachusetts Institute of Technology, Cambridge, MA, USA.; 2 Department of Electronics, Information and Bioengineering, Politecnico di Milano, Milan, ITA.; 3 Clinical Research Center, Artificial Intelligence Unit, Fundación Valle del Lili, Cali, Valle del Cauca, COL.; 4 University of California, Berkeley, CA, USA.; 5 University of Calabria, Rende, ITA.; 6 IDMEC, Instituto Superior Técnico, University of Lisbon, Lisbon, PRT.; 7 Department of Computer Science, National Tsing Hua University, Hsinchu, TWN.; 8 Department of Molecular Biosciences, The University of Texas at Austin, Austin, TX, USA.

## Abstract

Shortcut learning poses a significant challenge in clinical artificial intelligence, as models may rely on spurious signals rather than clinically relevant features, leading to biased predictions and poor generalization. Existing detection methods are fragmented and lack systematic evaluation across datasets and model architectures. To address this issue, we propose *ShortKit-ML*, an open-source Python framework for unified shortcut analysis in embedding spaces. The framework integrates over 20 detection methods and six mitigation strategies within a modular pipeline, encompassing embedding analysis, fairness metrics, training dynamics, causal methods, explainability, and representation analysis. We evaluate the framework on chest X-ray datasets (CheXpert and MIMIC-CXR), synthetic benchmarks, and an out-of-domain dataset (CelebA). Experimental results demonstrate that multi-method auditing provides more stable and interpretable evidence than individual methods, while detector disagreement reveals meaningful representational differences. The proposed framework offers automated reporting, interactive visualization, and is available as a pip-installable package. The source code and documentation are publicly available at https://github.com/criticaldata/ShortKit-ML and https://criticaldata.github.io/ShortKit-ML/.

## 1. Introduction

Deep learning has achieved substantial progress in medical imaging, with models reaching or exceeding expert-level performance across diverse diagnostic tasks [18, 40, 43]. However, accumulating evidence indicates that high predictive accuracy does not necessarily reflect clinically meaningful reasoning. Instead, models frequently exploit shortcuts: non-causal, task-irrelevant signals that are predictive within the training distribution but unstable under distribution shift [20]. In medical imaging, documented examples include chest drain markers used as proxies for pneumothorax [15], scanner identity predicting hip fracture labels with perfect accuracy [3], demographic attributes recoverable from radiology embeddings despite no direct clinical relevance [22], and, in organ segmentation, biases associated with sex, age, and scanner manufacturer [14].

The risks associated with shortcut learning, including patient safety and cross-institutional reliability, are amplified as large pre-trained and foundation models are increasingly deployed in clinical pipelines because shortcuts encoded in pre-trained representations can propagate to multiple downstream tasks. Beyond the concrete failure modes documented in the previous paragraph, broader empirical evidence has shown that diagnostic models can rely on institution-specific markers or acquisition artifacts rather than pathology [15, 58], and that models with stronger demographic encoding tend to exhibit larger subgroup performance disparities [8], whereas reduced demographic information has been associated with improved cross-institutional generalization [56].

Despite the importance of identifying shortcuts prior to deployment, existing detection methodologies remain fragmented. Fairness toolkits quantify group-level disparities [9], explainability methods visualize model attention patterns [47], and representation analyses probe embedding geometry [11, 50]. Moreover, prior work demonstrates that removing apparent bias components does not necessarily eliminate encoded structure; debiased embeddings may continue to cluster by protected attributes with high accuracy [25]. These approaches differ in assumptions, outputs, and operational interfaces, and are rarely combined within a standardized auditing workflow. As a result, researchers and practitioners must manually orchestrate heterogeneous analyses without a principled aggregation of evidence, which limits reproducibility and comprehensive evaluation. Moreover, in the absence of a proper characterization of representational bias, strategies such as oversampling, often used to “guarantee” balanced data, may fail to mitigate disparities and may even amplify pre-existing inequities [35].

In this work we present *ShortKit-ML*, a modular Python framework for systematic shortcut auditing in embedding spaces. The framework integrates over 20 detection methods and six mitigation strategies within a unified pipeline spanning six complementary paradigms: (1) embedding-level analysis, (2) fairness evaluation, (3) training dynamics, (4) causal and generative modeling, (5) explainability-driven techniques, and (6) representation analysis. Of these, 13 methods spanning five paradigms are systematically benchmarked in this work; the remaining seven require model internals (gradients, intermediate-layer activations), user-defined concept examples, or auxiliary model training and are therefore incompatible with an embedding-only evaluation protocol. The framework operates directly on vector representations, enabling systematic auditing without necessarily requiring access to model internals (important when working with black-box or API-served models). By aggregating heterogeneous detector outputs through a calibrated multi-perspective risk scoring mechanism, it reduces false positives and supports multi-attribute auditing and comparative benchmarking across model families. Although designed with clinical AI as the primary application, this embedding-level design is inherently domain-agnostic, as validated by the CelebA portability experiments in Section 4.1.3.

## 2. Related Work

Shortcut detection has developed across several largely parallel traditions, including shortcut learning, representation probing, fairness evaluation, slice discovery, and causal inference. We organize these lines of work here to clarify the methodological landscape that *ShortKit-ML* brings together.

Shortcut learning refers to models exploiting non-causal correlations that are predictive in the training distribution but unstable under distribution shift [20]. Canonical examples include texture bias in ImageNet classifiers [21] and annotation artifacts in natural language inference [39], with recent surveys formalizing broader taxonomies of spurious correlations and dataset bias [51,57]. In clinical AI, these shortcuts often arise from acquisition artifacts, scanner or institution identity, and demographic proxies. Prior work has shown, for example, that scanner model can strongly predict hip fracture labels [3], and that pathology and radiology models can rely on slide-origin metadata or visible artifacts such as chest drains, laterality markers, and rulers rather than clinically meaningful signal [10,15,29,46]. A complementary line of work asks what information is encoded in learned representations. Linear probes and related probing methods show that sensitive attributes are often recoverable from embeddings [1, 4], while geometric analyses reveal structured bias directions that may persist even after projection-based debiasing [11, 25]. Similar findings in vision embeddings further motivate auditing shortcuts directly in representation space rather than only through downstream predictions [50].

Fairness research in medical imaging has primarily emphasized subgroup performance disparities. Studies showing that chest X-ray models can predict patient race from images [22], along with reports of systematic error-rate disparities and subgroup degradation in foundation and vision-language models [24, 48, 55], have made demographic shortcut risk a central concern. However, this literature typically evaluates outcomes at the prediction level rather than identifying which components of the representation encode shortcut signals.

Related work on slice discovery and failure analysis instead examines structure within representation space. Methods such as GEORGE and Domino cluster embeddings to surface hidden subpopulations associated with systematic errors [19, 49], and related approaches use hierarchical clustering to identify failure regions [32]. These methods reveal latent heterogeneity, but they do not generally integrate fairness analysis, probing, and causal testing within a single workflow.

Statistical and causal approaches provide yet another perspective. Two-sample tests such as MMD and permutation-based testing quantify distributional differences across groups in embedding space [16,26], while methods such as Invariant Risk Minimization and counterfactual fairness seek representations that remain stable across environments or protected attributes [2,33]. These methods address important aspects of shortcut detection. However, they are usually applied in isolation.

Finally, several open-source toolkits support fairness auditing in practice, including Fairlearn, AIF360, and Aequitas [5,9,45]. These libraries standardize prediction-level fairness evaluation and mitigation, but do not provide integrated shortcut detection across representation, clustering, statistical, and causal perspectives.

Taken together, this literature shows that shortcut detection is inherently multi-dimensional, spanning representation geometry, subgroup disparities, latent clustering, statistical group differences, and invariance. However, despite the abundance of methods, they remain fragmented. *ShortKit-ML* is designed to address that gap by consolidating these complementary perspectives into a unified embedding-level auditing framework.

## 3 Framework Architecture

*ShortKit-ML* formulates shortcut detection in embedding space following prior taxonomy work on shortcut learning [51]. Let $E\in R^{n\times d}$ denote embeddings extracted from a trained model, with task labels Y and sensitive attributes A. We treat shortcut detection as identifying residual dependence between representations and sensitive attributes after conditioning on the task,

I(E;A∣Y)>0,

where I denotes conditional mutual information approximated through practical estimators. Intuitively, shortcut signals are present when embeddings remain separable by sensitive attributes beyond what is explained by the target labels. Because the framework operates directly on E, it is model-agnostic and independent of classifier architecture.

### 3.1. Detection Paradigms.

*ShortKit-ML* combines multiple partially independent estimators of conditional dependence, spanning 20 implemented methods across representation, fairness, learning-dynamics, perturbation, explainability, and latent-space analyses (full details in Appendix A). Geometry-based representation analysis includes linear and nonlinear probes, bias-aware clustering, dimension-wise statistical testing, bias-direction analysis, and centroid-based measures [1, 4, 6, 11, 26, 32]. Fairness modules quantify subgroup disparities using equalized odds, demographic parity, and intersectional analysis. Learning-dynamics modules capture shortcut-related training behavior through GroupDRO, generalized cross-entropy, and early-epoch clustering [42, 44]. Perturbation and counterfactual modules test stability under shortcut-targeted transformations, including frequency-based perturbations, causal-effect estimation, and generative counterfactual interventions [2, 53]. Explainability modules assess whether shortcut evidence is localized in clinically implausible regions using CAV, SpRAy, GradCAM overlap, and sufficient input subsets [34, 47]. Additional latent-space modules evaluate shortcut structure through disentanglement and spectral analysis.

### 3.2. Statistical Calibration and Aggregation.

Because these detector families produce heterogeneous outputs, *ShortKit-ML* calibrates each method before aggregation. Statistical tests are corrected for multiple comparisons using Bonferroni, Holm, and FDR-based procedures [6]; probe outputs are compared against empirical chance thresholds; and clustering outputs are normalized by cluster-quality confidence. Benchmark estimates across seeds are accompanied by bootstrap confidence intervals. In addition, each experimental configuration is evaluated on null synthetic embeddings with zero shortcut effect to estimate empirical false-positive rates and verify calibration under increasing dimensionality and multiple comparisons.

After calibration, each detector produces a normalized evidence score $s_{m}\in [0,1]$. Aggregated shortcut risk is then defined as

$$R=\frac{1}{\left|ℳ\right|}\sum_{m\in ℳ}⊮\left[s_{m}>\tau_{m}\right],$$

where $\tau_{m}$ is a method-specific significance threshold. Rather than relying on any single detector, the framework flags shortcuts through cross-paradigm agreement, which reduces false positives driven by artifacts specific to one methodological family. The primary aggregation strategy is indicator-count thresholding, while alternative voting-, weighting-, and meta-model-based schemes are provided in Appendix B.

### 3.3. Mitigation and Evaluation Loop.

The framework also includes data-level and model-level mitigation modules designed to reduce conditional dependence between embeddings and sensitive attributes [17, 31, 51, 59]. Data-level interventions include shortcut masking and background randomization, whereas model-level interventions include adversarial debiasing, explanation regularization, last-layer retraining, and contrastive debiasing. Mitigation effectiveness is assessed by rerunning the same detection pipeline on modified embeddings, enabling direct pre/post comparison under identical auditing criteria.

### 3.4. Modular Implementation.

Each detector implements a common interface,

$$\text{detect}(E,Y,A)\to s_{m},$$

which decouples individual analyses from the aggregation layer and allows new methods to be added without changing the overall workflow. *ShortKit-ML* is implemented in Python as a pip-installable package and supports embedding-only auditing, multi-attribute analysis, model comparison, and automated report generation. Additional implementation details, detector specifications, thresholds, and software components are provided in the Appendix.

## 4. Experiments

We evaluated *ShortKit-ML* in three settings: controlled synthetic benchmarks with known shortcut ground truth, two clinical chest X-ray cohorts to assess robustness across institutions and backbone families, and CelebA as a non-clinical external validation set. Together, these benchmarks test validity under controlled conditions, robustness within the clinical domain, and portability beyond it. Reproducibility details, including random seeds and preprocessing, are provided in Appendix D.

### 4.1. Experimental Design.

#### 4.1.1. Synthetic Benchmarks.

Synthetic embeddings were generated by designating a sparse subset of dimensions as shortcut-carrying and shifting them by ±δ (Cohen’s d) between demographic groups, while the remaining dimensions were sampled from a standard normal distribution. This design provides known shortcut locations and effect sizes, enabling direct comparison between detected and true shortcut dimensions. We varied four factors: effect size (δ∈{0.0,0.2,0.5,0.8,1.2,2.0}), sample size (n∈{200,1000,5000}), class imbalance ({0.5, 0.7, 0.9}), and embedding dimensionality (d∈{128,256,512}). Each configuration was repeated over 10 seeds.

Of the 20 detection methods implemented in *ShortKit-ML*, we benchmark the 13 that operate directly on embeddings without requiring model internals (gradients, intermediate-layer activations), user-defined concept examples (as needed for CAV), or auxiliary model training (as needed for VAE and CVAE counterfactual methods). These 13 embedding-native detectors span representation, fairness, and learning-dynamics families; 12 of the 13 were evaluated in the synthetic setting because intersectional analysis requires at least two sensitive attributes simultaneously. A detector was counted as positive when it met its pre-specified criterion after the corresponding correction or thresholding step (Appendix C). Performance was assessed using precision, recall, and F1-score for recovery of the true shortcut dimensions.

To evaluate false-positive control, we analyzed the null case (δ=0) and report per-method and aggregated false-positive rates under Bonferroni and FDR-BH correction. To evaluate robustness, we performed sensitivity analyses at δ=0.8, a moderately strong but non-saturated regime, while varying sample size, imbalance, and embedding dimensionality.

#### 4.1.2. Clinical Case Studies.

We next evaluated *ShortKit-ML* on two chest X-ray benchmarks. CheXpert [28] was studied using seven backbones (ResNet-50, DenseNet-121, ViT-B/16, ViT-B/32, DINOv2, RAD-DINO, and MedSigLIP) with 2,000 samples. MIMIC-CXR was studied using four backbones (RAD-DINO, ViT-B/16, ViT-B/32, and MedSigLIP) with approximately 1,491 samples. Three backbones (RAD-DINO, ViT-B/16, and MedSigLIP) were shared across datasets, enabling direct cross-dataset comparison. Pre-extracted embeddings and benchmark data are publicly available at https://huggingface.co/datasets/MITCriticalData/ShortKIT-ML-data.

We evaluated three sensitive attributes—sex, age (binarized as ≥60 vs <60, a threshold widely used in critical care cohort stratification), and race—and used agreement across the 13-method suite as the primary summary statistic. We chose agreement because no single detector is uniformly reliable across all shortcut geometries; cross-method convergence provides a more conservative summary in the presence of detector-specific false positives and blind spots. In addition to full-cohort analyses, we performed diagnosis-stratified analyses to test whether demographic encoding persists within clinically meaningful subgroups. Additional cohort statistics and per-diagnosis details are provided in the Appendix. Importantly, three methods—Bias Direction (PCA), SIS, and Demographic Parity—exhibit FPR=1.0 on null data across all tested embedding dimensionalities (Table 9); practitioners should default to the adjusted agreement counts that exclude these three methods (adjusted denominator: 10) when interpreting clinical results.

#### 4.1.3. CelebA Validation.

Finally, we evaluated *ShortKit-ML* on a non-clinical benchmark to assess generalization beyond medical imaging. We extracted 2,048-dimensional ResNet-50 embeddings from 10,000 CelebA images [37]. CelebA was selected because demographic shortcuts in facial representations are well documented and differ qualitatively from those in radiology. Rather than treating each CelebA label as an independent sensitive attribute (as in the clinical setting), we use Male as the single sensitive attribute A and test whether three CelebA binary labels that are known to be strongly correlated with Male—Blond Hair, Heavy Makeup, and Attractive [37]—are detectable as shortcut signals in the embedding space. This design tests whether the framework recovers well-documented gendered correlations in CelebA, making it a useful cross-domain validation of the detection pipeline rather than a demographic auditing exercise.

### 4.2. Experimental Results.

#### 4.2.1. Synthetic Results.

**Figure 1.**
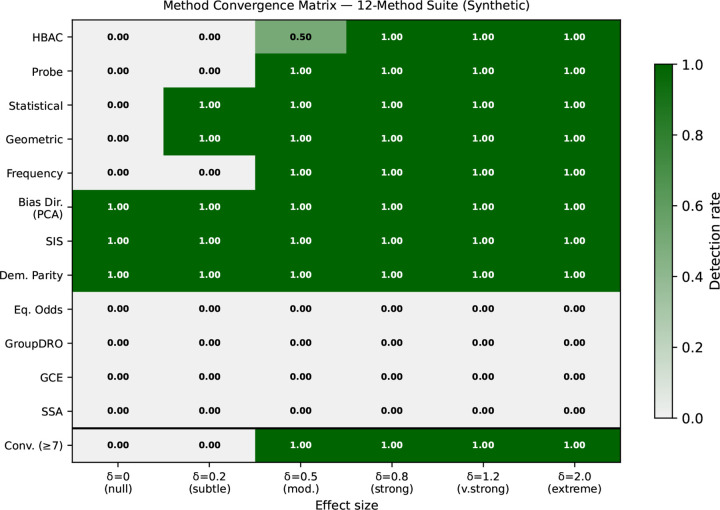
Convergence matrix across synthetic effect sizes (n=1000,d=128) for all 12 benchmarked methods (intersectional excluded from synthetic evaluation). Cell color: detection rate (1.0 = dark green, 0.0 = light gray). At δ=0, three methods (Bias Dir., SIS, Dem.Parity) flag on null data (high individual FPR) but HIGH-agreement convergence (≥7/12) remains FP=0.0. Agreement grows steadily: 5/12 at δ=0.2, 7–8/12 at δ=0.5, and plateaus at 8/12 for δ≥0.8.

The synthetic benchmark provides a controlled test of whether agreement across methods tracks shortcut strength and whether convergence improves reliability. Figure 1 summarizes agreement as the effect size increases across the full 12-method synthetic suite (13 benchmarked methods minus intersectional, which requires multi-attribute data). We separate two notions throughout this section: *raw agreement* is the number of methods (out of 12) that individually flag a configuration, while *convergence-based decisions* are the binary outcomes of fixed aggregation thresholds (e.g. HIGH = ≥7/12). The two are reported separately to avoid conflating method counts with aggregation outputs. Three patterns are evident. First, raw agreement rises with shortcut strength and then plateaus: it grows from 5/12 at δ=0.2 to 7–8/12 at δ=0.5 and remains at 8/12 from δ=0.8 onward (no further methods flag at δ=1.2 or δ=2.0). The four methods that never flag in the synthetic setting—Equalized Odds, Group-DRO, GCE, and SSA—account for the gap between 8/12 and 12/12; they rely on training-time loss dynamics or downstream prediction structure that is absent from the synthetic embeddings by construction (see also Tables 3, 9). Second, aggregated decisions are more stable than individual detectors in the low-signal regime: HIGH-agreement convergence (≥7/12) achieves zero false positives at all embedding dimensions despite three individual methods exhibiting 100% FPR on null data. Third, convergence improves false positive control: Indicator Count has FP=1.0 at δ=0 (driven by Bias Dir., SIS, Dem.Parity), whereas ≥7/12 reduces this to 0.0. Agreement reaches 5/12 at δ=0.2 and 8/12 at δ≥0.5. Detailed false positive rates, sensitivity analyses, and aggregation comparisons are reported in Appendix F. Results on harder synthetic variants (correlated and distributed shortcuts) are provided in Appendix F.1.

#### 4.2.2. Clinical Results.

The clinical experiments show that demographic encoding is detectable in both institutions and across multiple model families. On CheXpert, Table 4 shows that sex is detected with 6–8/13 method agreement across all seven backbones (3–5/10 excluding three methods—Bias Dir., SIS, Dem.Parity—with FPR=1.0 on null data; Table 9), while age and race are detected with 6–7/13 agreement (3–4/10 adjusted). On MIMIC-CXR (Table 5), agreement is slightly lower but remains consistent across models, with sex reaching 5–6/13 (2–3/10 adjusted) and age and race reaching 5/13 (2/10 adjusted). The three shared backbones between the datasets lead to the same qualitative conclusion in both cohorts: demographic information remains detectable regardless of whether the encoder is RAD-DINO, ViT-B/16, or MedSigLIP. Thus, while the magnitude of agreement varies across datasets, the broader detection pattern is reproduced across institutions and shared architectures.

**Figure 2.**
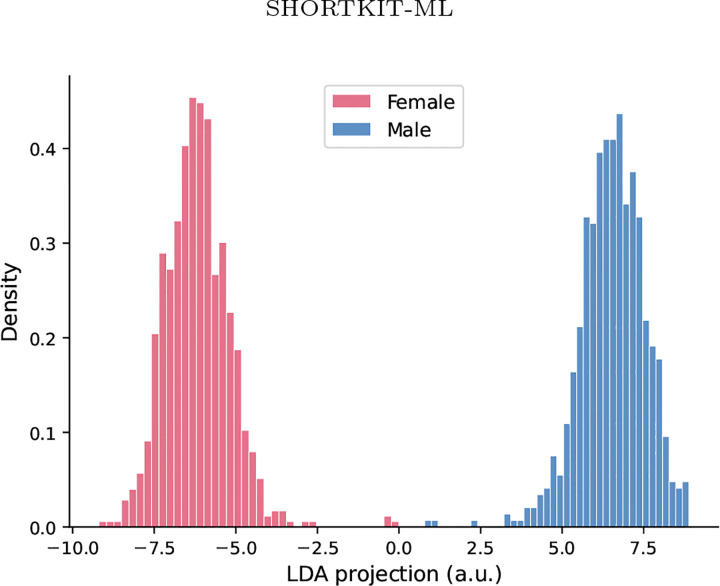
LDA projection of MIMIC-CXR RAD-DINO embeddings (768-dim, 1,491 samples) onto the maximally sex-discriminative direction. Nearcomplete separation confirms strong sex encoding in the representation space (probe F1=0.990; Table 13), consistent with 6/13 method agreement in Table 5.

A second observation is that the same subset of methods repeatedly contributes to detection. HBAC, frequency, bias direction PCA, SIS, and demographic parity tend to flag across many clinical settings, whereas statistical and geometric methods are consistently negative. A third, related observation: in both Tables 4 and 5, the per-method detection patterns for *age* and *race* are identical within every backbone, despite the two attributes having very different group structures (age is discretized into ordered bins, race is categorical with imbalanced group sizes). We do not interpret this as a positive finding about the attributes themselves; rather, it is a concrete example of how binary detection thresholds can flatten meaningful differences across attributes when the underlying signal sits in a similar range. This is a known limitation of indicator-count aggregation and is one reason we report convergence levels rather than raw per-method outputs as the primary summary in the discussion. Figure 2 illustrates this for MIMIC-CXR sex encoding, where an LDA projection confirms near-complete linear separability despite probe-only audits suggesting moderate risk. Additional embedding visualizations are provided in Appendix G. This pattern suggests that different detector families are sensitive to different forms of representational bias and motivates the use of a multi-paradigm framework rather than a single auditing tool.

#### 4.2.3. Per-Diagnosis Cross-Dataset Results.

We next examine whether the observed signals persist within clinically homogeneous subsets. Table 6 compares CheXpert and MIMIC-CXR after restricting analysis to patients positive for specific diagnoses. As expected, agreement is sometimes weaker than in the full-cohort analysis because diagnosis filtering reduces sample size. Even so, shortcut evidence remains visible across multiple diagnoses and across both datasets. This result suggests that the detected demographic signals are not explained solely by global cohort composition and persist within diagnosis-stratified subgroups.

#### 4.2.4. CelebA Results.

The CelebA experiments provide out-of-domain validation that the framework generalizes beyond medical imaging. Results in Table 7 show consistent shortcut detection across all three known gender-correlated attribute pairs (Blond Hair ↔ Male, Heavy Makeup ↔ Male, Attractive ↔ Male), with 10/13 methods agreeing on each (7/10 adjusted), substantially higher than the 5–8/13 observed on clinical data (2–5/10 adjusted).

The CelebA results are methodologically informative in two ways. GradCAM attention maps for a subset of CelebA samples are provided in Appendix I as supplementary visual evidence. First, learning-dynamics methods (GroupDRO, GCE, SSA) that produce no detections on the clinical benchmarks become active here, suggesting their sensitivity depends on the shortcut being visually salient and strongly encoded rather than subtle or distributed. Second, the high agreement across all three attributes is consistent with the framework not being tuned to medical-imaging-specific signal patterns; the same pipeline detects shortcuts in a fundamentally different domain and embedding space (ResNet-50, 2048-dim) without any modification. Together, these results support the modality-agnostic design claim and establish CelebA as a useful reference point for interpreting method behavior across domains.

## 5. Discussion

### 5.1. Demographic Encoding Is Consistent But Not Uniform.

This study introduces a framework for detecting and analyzing shortcuts in the embedding space. One of the main findings is that demographic information is consistently detectable in learned representations across datasets, backbone families, and analysis settings, but the form of that encoding is not uniform across domains or attributes. This shifts the question from whether shortcut information is present to how it is encoded and which classes of detectors are able to recover it reliably.

### 5.2. Encoding Persists Beyond Pathology.

A central concern in the clinical setting is whether the observed demographic signal is merely a byproduct of pathology. Our results do not support a purely pathology-mediated explanation. Demographic detection persists after restricting analysis to diagnosis-matched cohorts, and the No Finding subset in MIMIC-CXR still shows nontrivial agreement despite the absence of major pathology labels. The homogeneous cohort analysis in Table 2 strengthens this point: mean agreement remains 6.6/13 for sex and 6.3/13 for race on CheXpert, and 7.0/13 for sex on MIMIC-CXR (adjusted: 3.6/10, 3.3/10, and 4.0/10 respectively, excluding three always-flagging methods). While raw counts appear robust, adjusted counts indicate moderate rather than strong convergence; nevertheless, non-zero adjusted agreement across multiple backbones and diagnosis subgroups is consistent with genuine demographic encoding. We deliberately do not call these effects “not borderline”: the adjusted counts are moderate, and the strength of the conclusion depends on which exclusion set is applied. These results are consistent with the interpretation that pathology may contribute to demographic detectability, but does not fully explain it; demographic information appears to persist even within clinically narrower subgroups, though a causal account would require controlled intervention beyond the scope of this study.

From a clinical deployment perspective, this finding matters for any institution considering using these representations off-the-shelf. If demographic encoding persists after restricting to the same diagnosis, it means the problem is not simply solved by curating a diagnostically homogeneous training set. A model deployed for pleural effusion detection at a new hospital is likely to carry demographic structure learned during pretraining—one plausible interpretation of our findings is that careful curation of fine-tuning labels alone may be insufficient to remove this structure. These findings are consistent with the possibility that some portion of the persistent signal reflects anatomical or physiological correlates of demographic attributes that are not explicitly captured by diagnostic labels (for instance, sex-linked differences in chest anatomy). We make this observation cautiously: it does not justify inferring biological causation from embedding statistics, and we do not extend the claim to race or to socially defined attributes, where institutional, acquisition, and labeling pathways are more plausible drivers (Section 5).

### 5.3. Detector Family Behavior Reveals Encoding Geometry.

Another important question is how this information is represented. Here, the behavior of different detector families becomes informative. The probe results reveal one clear limitation of probe-only auditing: in the clinical benchmarks, probes detect sex with high separability but fail to detect race, even though several other method families repeatedly indicate that race information is present in the embeddings. This contrast suggests that sex is encoded in a relatively linear and accessible form, whereas race is encoded through weaker, more distributed, or nonlinear structure. In practical terms, an audit pipeline based primarily on linear probes could conclude that a representation is safe with respect to race while missing a signal that is present but not linearly separable. More broadly, this difference across methods suggests that disagreement should not be treated simply as noise. When HBAC, bias-direction methods, and fairness-based criteria flag consistently while probes or learning-dynamics methods do not, the disagreement reflects something about the geometry of the representation itself. Some attributes appear linearly accessible, others directionally distributed, and others detectable only through changes in group-conditioned behavior. In that sense, *ShortKit-ML* is useful not only as a binary auditing tool but also as a way of characterizing representational structure. The framework helps distinguish not just whether sensitive information is present, but also the form in which it is encoded. This also adds practical value to development pipelines in applied settings, as it enables the selection of auditing and mitigation strategies that are better aligned with how bias is encoded in the representation.

### 5.4. Learning-Dynamics Methods Are Domain-Dependent.

This interpretation also helps explain the domain dependence of the learning-dynamics methods. GCE detects all three known shortcuts on CelebA and reaches 10/13 agreement there, yet it does not flag any attributes across the clinical benchmark. GroupDRO and SSA show a similar lack of sensitivity. The most plausible explanation is that minority-loss and reweighting signals become informative when shortcut features are strong, visually salient, and tightly coupled to downstream prediction behavior, as in facial images. In chest X-ray embeddings, by contrast, the demographic signal appears weaker, more distributed, or less directly reflected in the loss dynamics of the downstream model. The implication is not that learning-dynamics methods are unhelpful, but that they are unlikely to be sufficient on their own for medical representation auditing. Their value appears to depend strongly on the strength and geometry of the underlying shortcut.

### 5.5. Shortcut Strength Depends on Institution and Cohort.

Finally, the No Finding analysis makes a related point from a different angle. Because this subset reduces the role of overt pathology, it offers a useful stress test of whether shortcut detection survives outside obvious disease-linked structure. Here the two datasets differ substantially: CheXpert shows weak sex agreement (1/13), whereas MIMIC-CXR shows stronger agreement (5/13). Cohort statistics in Appendix G suggest that this divergence is likely related to differences in demographic composition, subgroup balance, labeling practice, or acquisition context. Rather than weakening the framework, this result underscores an important observation: shortcut strength is not an intrinsic property of the imaging modality alone, but is shaped by the institution, the cohort, and the data-generation process. At this level, institutional cultures of data governance and standardization also play a role, as do operational practices and even individual decisions that may introduce or perpetuate biases across subgroups. This suggests that many of these patterns do not originate exclusively in the model, but may already be present from the way the data are structured. At the same time, the results are not arbitrary. Across CNN, transformer, self-supervised, and vision-language backbones, the same broad detection patterns recur. We do not claim that architecture is irrelevant. Rather, within the evaluated backbone families, demographic signals appear preserved despite substantial differences in model design and pretraining strategy. This pattern is more consistent with a data-distribution explanation than an architecture-specific one, though the current evidence does not permit causal attribution. Practically, this suggests that changing the encoder alone may be insufficient to remove demographic encoding if the training data continue to carry that structure.

### 5.6. Why Detectors Disagree: Shortcuts as Compressed Heuristics.

The following offers one conceptual interpretation of the observed detection patterns; it is a hypothesis rather than an established mechanistic account. Probes recover sex encoding from most chest X-ray embeddings, but all miss race. Clustering and fairness methods flag both. GCE, detecting all 3 shortcuts on CelebA, produces no signal on clinical embeddings, despite demographic structure being present by other measures. GroupDRO and SSA show no sensitivity in either domain. The same framework applied to the same modality at two institutions yields different agreement levels. Why?

Gradient descent, like natural selection, converges on the cheapest solution its environment offers. When a training distribution contains a regularity that costs less to exploit than the true causal mechanism, the model will learn it — not because the optimizer is defective, but because it is doing exactly what it was designed to do. A shortcut is a compressed decision rule that exploits environmental regularity to reduce processing cost. This holds whether the system is a neural network learning to associate laterality markers with COVID-19 classification or an organism developing a foraging heuristic that conserves calories in a specific ecology [23, 52].

Different detectors are sensitive to different forms of compression. A linear probe recovers attributes encoded along accessible linear directions — the embedding equivalent of a simple, high-fidelity heuristic. Sex has strong anatomical correlates visible in chest radiographs; one plausible reason it is more linearly accessible than race is that such correlates provide a consistent, high-amplitude encoding signal. Race enters representations through subtler, more distributed pathways: institutional acquisition patterns, demographic composition of training cohorts, correlations between scanner characteristics and patient populations. Its encoding is correspondingly less linear. Clustering methods and fairness metrics, which impose no linearity assumption, recover it; probes do not. Detector disagreement is not noise; it provides evidence about the underlying geometry of the shortcut.

The same logic accounts for domain dependence. GCE detects shortcuts on CelebA because facial gender encoding is strong, visually salient, and tightly coupled to the loss surface — an efficient, high-fidelity compression. In chest X-ray embeddings, demographic signals are weaker, more distributed, less directly reflected in loss-landscape structure, and therefore invisible to methods that rely on it. The shortcut is present in both domains; the form of compression differs, and so does the set of detectors capable of recovering it.

Institutional variation reinforces the point from a different angle. Across biological and cultural systems, heuristics are ecologically rational: adaptive within the environments that shaped them, unreliable when exported [7]. Standards derived from one population do not generalize to another [27]; decision rules calibrated to one ecology become maladaptive under mismatch [36]. Computational shortcuts follow the same logic. A model trained at one institution learns the statistical regularities of that institution’s acquisition pipeline, patient demographics, and labeling practices. Deployed at a second institution, those regularities no longer hold, and the shortcut becomes a source of systematic error. The divergence between CheXpert and MIMIC-CXR on the No Finding subset — 1/13 versus 5/13 sex agreement — is consistent with this: the shortcut is shaped by the data-generating environment, not solely by the imaging modality.

The question “is a shortcut present?” is therefore incomplete. It needs a companion: in what form is it encoded, and in what deployment context will it cause harm? The critical variable is not the shortcut itself but the match between the compressed regularity and the environment where the model operates. A shortcut is adaptive when the regularity it exploits is causally grounded and matched to the deployment context; it becomes harmful when either condition fails. Multi-perspective auditing is not a statistical convenience. It is a structural necessity imposed by the nature of the phenomenon: because shortcuts vary in their encoding geometry, no single detector family can recover all of them.

A full epistemological treatment of shortcuts across biological and computational substrates, including questions of causal relevance, cultural mediation, and contextual legitimacy, is the subject of future work.

### 5.7. Framework Scope and Clinical Deployment Implications.

Taken together, these findings clarify the scope and utility of the framework. *ShortKit-ML* is designed to operate directly on vector representations, enabling modality-agnostic auditing without access to model internals. The three use cases in this paper, synthetic benchmarks, clinical chest X-ray embeddings, and CelebA face images, serve as demonstrations of framework utility under different shortcut regimes, not as exhaustive empirical claims about demographic encoding in general. Portability to text, audio, and multimodal embeddings is an architectural property of the framework; validating it empirically remains future work. Even so, the case studies support a broader principle: shortcut auditing should not be treated simply as a yes-or-no question, but as a structural, cultural, and representational understanding problem, where the way data are produced, organized, and used influences how biases become encoded, and in which detector complementarity and calibrated convergence provide richer evidence than any single metric.

## 6. Limitations and Future Work

Several limitations should be acknowledged. First, the framework assumes access to labeled sensitive attributes. In practice, such labels may be unavailable, incomplete, or socially imperfect proxies. Semi-supervised components such as SSA partly relax this requirement, but they do not eliminate it. Relatedly, race binarization in MIMIC-CXR (White vs. non-White, collapsing Black, Asian, Hispanic, and Other into a single group) is an aggressive simplification driven by sample-size constraints; it likely underestimates within-group heterogeneity and may suppress detection of encoding patterns specific to individual racial groups, a concern particularly relevant for fairness-oriented applications. Second, some interpretation thresholds, such as using 70% probe accuracy as a high-risk indicator, are empirically useful in our experiments but may require domain-specific calibration [4]. Third, the geometric analysis method produces false positive rates of 1.00 at embedding dimensionalities d≥256 on null data (Table 9); it should therefore only be used within a convergence-based pipeline rather than as a standalone detector in high-dimensional settings. The same applies, and more broadly, to Bias Direction (PCA), SIS, and Demographic Parity, which produce FPR=1.0 at all tested dimensionalities (d=128,256,512), and to Frequency, which reaches FPR=1.0 at d=512 (Table 9). Clinical agreement counts in this paper include these methods; adjusted counts excluding the three universal false-positive methods are lower but remain non-zero (Section 4). Fourth, the full framework is computationally nontrivial: running the complete 13-method suite may be burdensome on very large datasets, so practitioners may need to select subsets of methods depending on the audit objective. Fifth, explainability-based detectors such as SIS are less effective for non-local or spatially variable shortcuts. For example, ECG leads in chest X-rays may indicate patient monitoring status, but their visual appearance varies across patients and acquisition settings, making them difficult to localize consistently with attribution methods. Sixth, the notion of a “shortcut” is not entirely context-free. Cultural norms, institutional practices, and population composition can all affect which representational patterns are considered problematic or benign [7,23,27,36,52]. For that reason, detection results should be interpreted within the clinical and social context in which the model will be deployed. Importantly, detection of sensitive attribute encoding in embeddings does not necessarily imply causal reliance by the model or measurable downstream harm in prediction performance. Rather, it should be interpreted as an indicator of potential shortcut risk that warrants further investigation.

Several directions for future work follow naturally from these limitations. One is extension to multimodal embeddings, especially vision-language models, where demographic bias has already been reported in clinical AI [55]. Another is to replace simple agreement counts with calibrated ensemble statistics, such as weighted voting or confidence intervals over convergence. A third is to improve scalability through GPU acceleration and incremental computation. Finally, the field would benefit from standardized benchmarks, ideally with partial or full shortcut ground truth, building on efforts such as MEDFAIR [60] and FairMedFM [30]. For *ShortKit-ML* specifically, the most immediate next step is calibration of convergence thresholds and ensemble aggregation rules, since that would strengthen both interpretability and statistical reliability in real-world audits.

## 7. Conclusion

We presented *ShortKit-ML*, a modular framework for shortcut auditing in clinical AI embedding spaces. The main finding is that shortcut signals are more reliably identified through calibrated agreement across complementary detectors than through any single method alone. This pattern held across synthetic benchmarks with known ground truth, across clinical chest X-ray cohorts and backbone families, and in out-of-domain validation on CelebA. By unifying multi-method auditing within a single embedding-level workflow, *ShortKit-ML* makes representation auditing more practical for clinical AI research and deployment. As foundation models are increasingly integrated into clinical pipelines, such auditing will be an important component of responsible use.

## Figures and Tables

**Table 1.** Comparison of existing bias and shortcut detection tools.

Tool	Level	Embedding Analysis	Modality	Multi-Method	Auto Report	Medical Focus
Fairlearn [9]	Prediction	No	Tabular	No	No	No
AIF360 [5]	Prediction	No	Tabular	Partial	No	No
Aequitas [45]	Prediction	No	Tabular	Partial	Yes	No
What-If Tool [54]	Prediction	No	Tabular	Yes	Yes	No
WEAT [12]	Embedding	Word only	Text	No	No	No
Domino [19]	Embedding	Slice discovery	Vision/Text	No	No	No
MEDFAIR [60]	Prediction	No	Vision (med.)	Partial	No	Yes
FairMedFM [30]	Prediction	No	Vision (med.FMs)	Partial	No	Yes
***ShortKit-ML* (Ours)**	**Embedding**	**Yes (20+)**	**Vision/Text/Tab.**	**Yes**	**Yes**	**Yes**

**Table 2.** Homogeneous cohort analysis: demographic encoding within diagnosis-matched subgroups across 3 shared backbones (RAD-DINO, MedSigLIP, ViT-B/16) and 2 datasets. Mean agreement across backbones shown. All values ≥5/13, consistent with direct demographic encoding independent of pathology. Adjusted values excluding three always-flagging methods (Bias Dir., SIS, Dem. Parity; FPR = 1.0 on null data) range from approximately 2–4/10. In this table, the “minority group” is defined per attribute as the smaller of the two label classes within the diagnosis subset (Female for sex; Non-White (Black/Asian/Other collapsed) for race), following the binary encoding used in Table 13. † The dagger marks rows where the minority-group sample count within the diagnosis subset falls below 20 and probe/clustering estimates become unreliable.

Diagnosis	CheXpert		MIMIC-CXR	
	Sex	Race	Sex	Race
Lung Opacity	6.7/13	6.0/13	7.3/13	†
Pleural Effusion	6.7/13	6.0/13	6.7/13	†
Support Devices	6.7/13	7.3/13	6.7/13	†
Atelectasis	6.7/13	†	7.3/13	†
Edema	6.3/13	5.7/13	7.3/13	†
**Mean**	**6.6/13**	**6.3/13**	**7.0/13**	†

**Table 3.** Synthetic benchmark: detection rate (Det.) and localization F1 (where applicable) across effect sizes (n=1000,d=128, 10 seeds) for all 12 benchmarked methods, grouped by tier. δ=0 (null) data available only for the Tier 1 core subset (†). Learning-dynamics methods (Tier 3) do not flag in the synthetic setting across all δ, consistent with their reliance on trainingtime loss dynamics rather than embedding geometry. ★Geometric FP rate reaches 1.00 at d≥256 on null data; should only be used within a convergencebased pipeline. Intersectional analysis excluded from the synthetic benchmark (requires ≥2 sensitive attributes simultaneously).

Method	Met.	δ=0 ^†^	δ=0.2	δ=0.5	δ=0.8	δ=1.2	δ=2.0
*Tier 1* — *Embedding-Native*							
HBAC	Det.	0.00	0.00	0.50	1.00	1.00	1.00
	F1	.04±.08	.04±.08	1.0±.00	1.0±.00	1.0±.00	1.0±.00
Probe	Det.	0.00	0.00	1.00	1.00	1.00	1.00
	F1	.00±.00	.98±.06	1.0±.00	1.0±.00	1.0±.00	1.0±.00
Statistical	Det.	0.40	1.00	1.00	1.00	1.00	1.00
	F1	.00±.00	1.0±.00	1.0±.00	1.0±.00	1.0±.00	1.0±.00
Geometric^★^	Det.	0.00	1.00	1.00	1.00	1.00	1.00
	F1	.00±.00	1.0±.00	1.0±.00	1.0±.00	1.0±.00	1.0±.00
Frequency	Det.	—	0.00	1.00	1.00	1.00	1.00
Bias Dir. PCA	Det.	—	1.00	1.00	1.00	1.00	1.00
	F1	—	1.0±.00	1.0±.00	1.0±.00	1.0±.00	1.0±.00
SIS	Det.	—	1.00	1.00	1.00	1.00	1.00
	F1	—	.84±.16	.98±.06	.94±.10	.88±.14	.50±.11
*Tier 2* — *Fairness-Based*							
Dem. Parity	Det.	—	1.00	1.00	1.00	1.00	1.00
Equal. Odds	Det.	—	0.00	0.00	0.00	0.00	0.00
*Tier 3* — *Learning-Dynamics*							
GroupDRO	Det.	—	0.00	0.00	0.00	0.00	0.00
GCE	Det.	—	0.00	0.00	0.00	0.00	0.00
SSA	Det.	—	0.00	0.00	0.00	0.00	0.00
Conv. (≥7/12)	Det.	0.00	0.00	1.00	1.00	1.00	1.00

**Table 4.** CheXpert 13-method shortcut detection results across 7 backbone architectures and 3 sensitive attributes (2,000 samples each). ✓ = detected, ⋅ = not detected. Sex shortcuts reach 8/13 agreement on general-purpose backbones (ResNet-50 through DINOv2) and 6–7/13 on domain-specific models (RAD-DINO, MedSigLIP). Age and race are consistently detected at 6–7/13. Statistical and geometric methods never flag, while HBAC, frequency, bias direction PCA, SIS, and demographic parity consistently flag across all configurations. These three methods have FPR=1.0 on null data at all tested dimensionalities (Table 9); adjusted agreement excluding them ranges from 3–5/10. Within every backbone, the per-method detection vector for Age and Race is identical because both attributes fall within the same indicatorcount bin under the fixed thresholds in Appendix C; this is a consequence of binary thresholding rather than evidence that age (ordered bins) and race (categorical) are encoded identically. The same pattern recurs in Table 5 and is discussed in Section 4.2.

Backbone	Attribute	HBAC	Probe	Stat.	Geom.	Freq.	BiasDir	SIS	DP	EO	Inter.	GDRO	GCE	SSA	Agree.
ResNet-50	Sex	✓	✓	·	·	✓	✓	✓	✓	✓	✓	·	·	·	8/13
	Age	✓	·	·	·	✓	✓	✓	✓	✓	✓	·	·	·	7/13
	Race	✓	·	·	·	✓	✓	✓	✓	✓	✓	·	·	·	7/13
DenseNet-121	Sex	✓	✓	·	·	✓	✓	✓	✓	✓	✓	·	·	·	8/13
	Age	✓	·	·	·	✓	✓	✓	✓	✓	✓	·	·	·	7/13
	Race	✓	·	·	·	✓	✓	✓	✓	✓	✓	·	·	·	7/13
ViT-B/16	Sex	✓	✓	·	·	✓	✓	✓	✓	✓	✓	·	·	·	8/13
	Age	✓	·	·	·	✓	✓	✓	✓	✓	✓	·	·	·	7/13
	Race	✓	·	·	·	✓	✓	✓	✓	✓	✓	·	·	·	7/13
ViT-B/32	Sex	✓	✓	·	·	✓	✓	✓	✓	✓	✓	·	·	·	8/13
	Age	✓	·	·	·	✓	✓	✓	✓	✓	✓	·	·	·	7/13
	Race	✓	·	·	·	✓	✓	✓	✓	✓	✓	·	·	·	7/13
DINOv2	Sex	✓	✓	·	·	✓	✓	✓	✓	✓	✓	·	·	·	8/13
	Age	✓	·	·	·	✓	✓	✓	✓	✓	✓	·	·	·	7/13
	Race	✓	·	·	·	✓	✓	✓	✓	✓	✓	·	·	·	7/13
RAD-DINO	Sex	✓	✓	·	·	✓	✓	✓	✓	·	✓	·	·	·	7/13
	Age	✓	·	·	·	✓	✓	✓	✓	·	✓	·	·	·	6/13
	Race	✓	·	·	·	✓	✓	✓	✓	·	✓	·	·	·	6/13
MedSigLIP	Sex	✓	·	·	·	✓	✓	✓	✓	·	✓	·	·	·	6/13
	Age	✓	·	·	·	✓	✓	✓	✓	·	✓	·	·	·	6/13
	Race	✓	·	·	·	✓	✓	✓	✓	·	✓	·	·	·	6/13

**Table 5.** MIMIC-CXR 13 -method shortcut detection results across 4 backbone architectures and 3 sensitive attributes (~1,491 samples for sex/age, ~1,273 for race). ✓= detected, ⋅ = not detected. Sex reaches 5–6/13 agreement; race and age reach 5/13 across all backbones. HBAC, bias direction PCA, and SIS form the consistent detection core that flags across all four backbones; Frequency joins them for RAD-DINO, ViT-B/16, and ViT-B/32 but does not flag on MedSigLIP, so it is part of the core for general-purpose backbones only. Bias Dir. and SIS have FPR=1.0 on null data (Table 9); adjusted agreement excluding the three highest-FPR methods ranges from 2–3/10.

Backbone	Attribute	HBAC	Probe	Stat.	Geom.	Freq.	BiasDir	SIS	DP	EO	Inter.	GDRO	GCE	SSA	Agree.
	Sex	✓	✓	·	·	✓	✓	✓	✓	·	·	·	·	·	6/13
RAD-DINO	Age	✓	·	·	·	✓	✓	✓	✓	·	·	·	·	·	5/13
	Race	✓	·	·	·	✓	✓	✓	✓	·	·	·	·	·	5/13
	Sex	✓	✓	·	·	✓	✓	✓	✓	·	·	·	·	·	6/13
ViT-B/16	Age	✓	·	·	·	✓	✓	✓	✓	·	·	·	·	·	5/13
	Race	✓	·	·	·	✓	✓	✓	✓	·	·	·	·	·	5/13
	Sex	✓	·	·	·	✓	✓	✓	✓	·	·	·	·	·	5/13
ViT-B/32	Age	✓	·	·	·	✓	✓	✓	✓	·	·	·	·	·	5/13
	Race	✓	·	·	·	✓	✓	✓	✓	·	·	·	·	·	5/13
	Sex	✓	✓	·	·	·	✓	✓	·	✓	✓	·	·	·	6/13
MedSigLIP	Age	✓	·	·	·	·	✓	✓	·	✓	✓	·	·	·	5/13
	Race	✓	·	·	·	·	✓	✓	·	✓	✓	·	·	·	5/13

**Table 6.** Cross-dataset per-diagnosis shortcut detection using 13 methods. H=HBAC, P=Probe, S=Statistical, G=Geometric, Fr=Frequency, BD=Bias Direction PCA, SI=SIS, DP=Demographic Parity, EO=Equalized Odds, In=Intersectional, GD=GroupDRO, GC=GCE, SS=SSA. ✓ = flagged, ⋅ = not flagged.

Diagnosis	n	H	P S		G	Fr	BD	SI	DP	EO	In	GD	GC	SS	Agr.
*CheXpert (sex)*															
Lung Opacity	827	✓	✓	·	·	✓	✓	✓	·	·	·	·	·	·	5/13
Atelectasis	266	✓	·	·	·	✓	✓	✓	·	·	·	·	·	·	4/13
No Finding	259	·	·	·	·	·	✓	·	·	·	·	·	·	·	1/13
Pleural Eff.	730	✓	✓	·	·	✓	✓	✓	·	·	·	✓	·	·	5/13
Support Dev.	933	✓	✓	·	·	✓	✓	✓	·	·	·	·	·	·	5/13
*MIMIC-CXR (sex)*															
Lung Opacity	305	✓	✓	·	·	✓	✓	✓	·	·	✓	·	·	·	6/13
Atelectasis	263	✓	✓	·	·	✓	✓	✓	✓	·	✓	·	·	·	7/13
No Finding	545	✓	✓	·	·	✓	✓	✓	·	·	·	·	·	·	5/13
Pleural Eff.	255	✓	✓	·	·	✓	✓	✓	✓	·	✓	·	·	·	7/13
Support Dev.	239	✓	✓	·	·	✓	✓	✓	✓	·	·	·	·	·	6/13
*MIMIC-CXR (race)*															
Lung Opacity	252	✓	·	·	·	✓	✓	✓	✓	·	·	·	·	·	5/13
Atelectasis	229	✓	·	·	·	✓	✓	✓	✓	·	·	·	·	·	5/13
No Finding	464	✓	·	·	·	✓	✓	✓	·	·	·	·	·	·	4/13
Pleural Eff.	224	✓	·	·	·	✓	✓	✓	✓	·	✓	·	·	·	6/13
Support Dev.	194	✓	·	·	·	✓	✓	✓	✓	·	·	·	·	·	5/13

**Table 7.** CelebA validation on real ResNet-50 embeddings (2,048-dim, 10,000 images) using all 13 methods. Known shortcuts (attribute correlations): blond hair, heavy makeup, and attractiveness are correlated with gender. All three known shortcuts are detected with 10/13 agreement. HBAC, GroupDRO, and SSA do not flag; GCE detects on CelebA, in contrast to clinical datasets where it flags 0/13. ✓ = detected, ⋅ = not detected. Bias Dir., SIS, and Dem. Parity flag on all three rows (FPR=1.0 on null data); adjusted agreement excluding these three methods is 7/10, confirming strong shortcut signal independent of always-flagging methods.

Shortcut Pair	HBAC	Probe	Stat.	Geom.	Freq.	BiasDir	SIS	DP	EO	Inter.	GDRO	GCE	SSA	Agree.
Blond_Hair ↔ Male	·	✓	✓	✓	✓	✓	✓	✓	✓	✓	·	✓	·	10/13
Heavy_Makeup ↔ Male	·	✓	✓	✓	✓	✓	✓	✓	✓	✓	·	✓	·	10/13
Attractive ↔ Male	·	✓	✓	✓	✓	✓	✓	✓	✓	✓	·	✓	·	10/13

**Figure 3.**
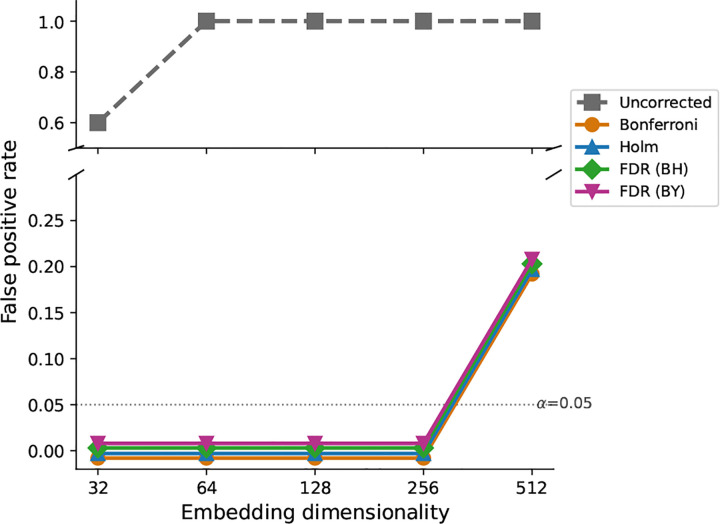
False positive rate of statistical testing on null data (δ=0) as a function of embedding dimensionality, comparing uncorrected testing against four multiple testing correction methods (Bonferroni, Holm, FDR-BH, FDR-BY). Uncorrected testing reaches 100% FPR at d≥64; all correction methods maintain FPR near 0% through d=256.

**Figure 4.**
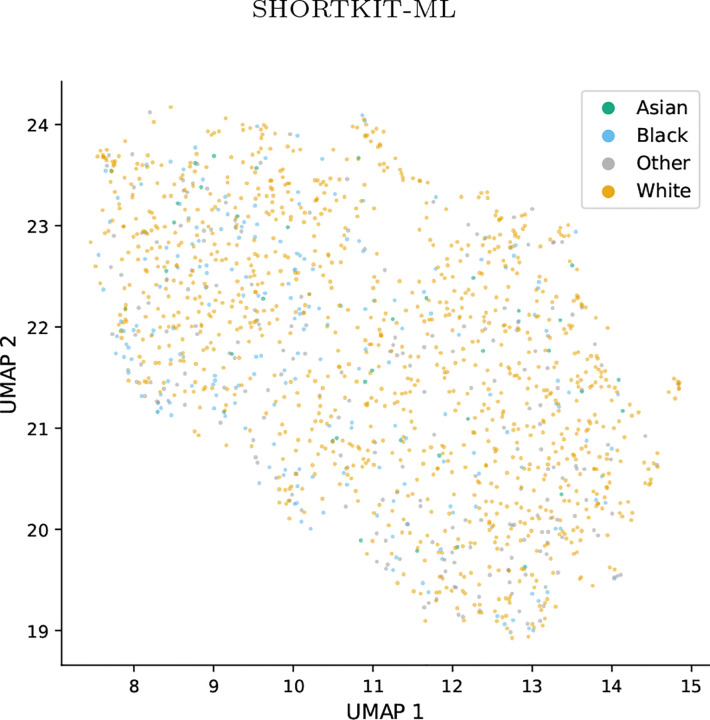
UMAP projection of MIMIC-CXR RAD-DINO embeddings colored by race (simplified: White, Black, Asian, Other). Race groups show partial overlap with some structure, consistent with 5/13 method agreement for race in Table 5.

**Figure 5.**
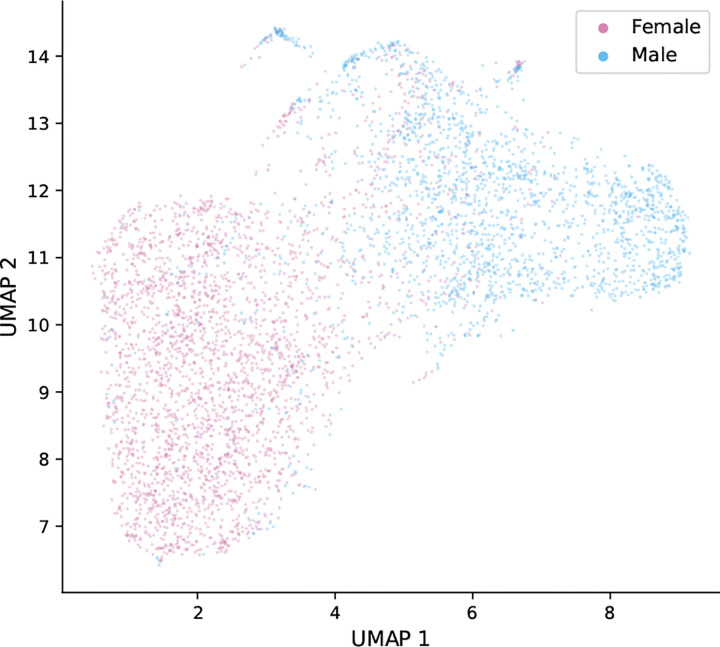
UMAP projection of CelebA ResNet-50 embeddings (2,048-dim, 5,000 subsample) colored by gender. Strong cluster separation confirms that gender is a dominant axis of variation in the embedding space, consistent with 94% probe F1 and 1,939/2,048 FDR-significant dimensions.

**Figure 6.**
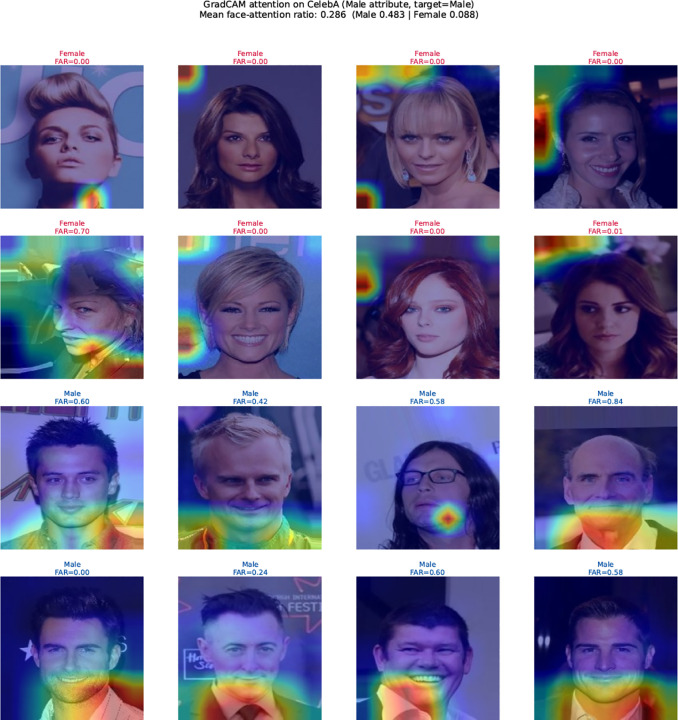
GradCAM attention overlays for 16 CelebA samples (8 Female top two rows, 8 Male bottom two rows). Each panel shows the original face image blended with the jet-coloured GradCAM heatmap (targeting the Male class). Face-attention ratio (FAR) is annotated per image. Male-predicted images show concentrated attention on facial regions (mean FAR=0.48), while Female-predicted images show diffuse attention (mean FAR=0.09).

## Appendix A. Detection Method Details

This appendix provides per-method descriptions for all 20 detection methods implemented in *ShortKit-ML*.

### A.1. Representation-Level Methods.

**HBAC (Hierarchical Bias-Aware Clustering)** detects whether embeddings naturally cluster according to sensitive attributes [32, 41]. Hierarchical clustering is applied to the embedding space and the purity of resulting clusters is measured with respect to sensitive attribute labels:

(1)
$$\text{Purity}(C)=\frac{{\text{max}}_{a}|\{x\in C:A(x)=a\}|}{\left|C\right|}$$

Additionally, linearity, the degree to which cluster boundaries align with linear hyperplanes, is measured. Purity > 80% combined with high linearity is consistent with shortcut presence.

**Probe-Based Detection** trains lightweight auxiliary classifiers (linear logistic regression or shallow MLPs) to predict A from E [1,4,22]. The framework provides both sklearn and PyTorch probe implementations. Probe accuracy > 85% is consistent with HIGH shortcut risk; 70%–85% suggests MODERATE risk; below 70% is LOW or absent.

**Statistical Testing** identifies which specific embedding dimensions encode group differences using dimension-wise hypothesis tests (Mann-Whitney U for binary, Kruskal-Wallis for multi-class). Four multiple testing correction methods are supported: Bonferroni, Holm stepdown, FDR-BH (default), and FDR-BY [6]. More than 30% of dimensions showing significant differences after correction is consistent with strong shortcut encoding.

**Geometric Analysis** leverages embedding geometry to find shortcut axes. Centroid distances between demographic groups are computed, and effect sizes are estimated. Large centroid separation relative to within-group variance indicates systematic encoding of group membership.

**Bias Direction PCA** extracts a bias direction from group prototypes (centroids) using PCA and measures the projection gap along this direction [11]. A large projection gap indicates that group identity is encoded along a dominant linear direction in the embedding space.

### A.2. Fairness Methods.

**Equalized Odds** evaluates TPR and FPR disparities across demographic groups. A lightweight classifier is trained on embeddings, and the maximum absolute difference in TPR and FPR across groups is reported.

**Demographic Parity** measures positive prediction rate differences across groups, flagging cases where the prediction rate varies substantially by sensitive attribute.

**Intersectional Analysis** evaluates fairness across intersections of demographic attributes (e.g., Black × Female), capturing compounded disparities that single-attribute analysis may miss.

### A.3. Training Dynamics Methods.

**GroupDRO** measures worst-group performance gaps using distributionally robust optimization [44]. The loss is upweighted for the worst-performing demographic group, and the gap between worst-group and average loss indicates shortcut reliance.

**GCE (Generalized Cross Entropy)** uses a generalized cross-entropy loss with tunable parameter q≈0.7 to identify high-loss samples that may belong to shortcut-disadvantaged minority groups [42].

**Early-Epoch Clustering (SPARE)** detects shortcut reliance in early-epoch representations by clustering intermediate embeddings and measuring whether group structure emerges before task-relevant structure [42].

### A.4. Perturbation and Counterfactual Methods.

**Frequency Analysis** removes frequency bands from embeddings and measures the change in downstream task performance, identifying shortcuts concentrated in specific frequency components [53].

**Causal Effect Estimation** identifies attributes with near-zero causal influence on predictions, building on invariant risk minimization [2] and causal reasoning for medical imaging [13].

**Generative CVAE** uses a conditional variational autoencoder to generate counterfactual embeddings (altering group membership while preserving other factors) and measures the resulting prediction or representation shift.

### A.5. Explainability Methods.

**CAV (Concept Activation Vectors)** tests whether learned representations are sensitive to user-defined concepts (e.g., “chest drain presence”) by training linear classifiers on concept examples and measuring directional derivatives.

**SpRAy** performs spectral clustering of GradCAM attribution heatmaps across a dataset, surfacing systematic attention patterns that may indicate Clever Hans strategies [34].

**GradCAM Mask Overlap** quantifies the spatial overlap between GradCAM attention maps and known shortcut regions (provided as binary masks) [47].

**SIS (Sufficient Input Subsets)** identifies minimal subsets of embedding dimensions that are sufficient for prediction, flagging cases where a small number of dimensions dominate.

### A.6. Representation Analysis Methods.

**VAE Disentanglement** trains a variational autoencoder on embeddings and evaluates whether latent factors separate by sensitive attributes, indicating entangled shortcut encoding.

**SSA (Semi-Supervised Spectral Analysis)** uses spectral methods on the embedding similarity graph to detect structure aligned with group labels, providing a semi-supervised approach that requires minimal labeled data.

## Appendix B. Risk Aggregation Conditions

The five pluggable risk conditions map calibrated evidence scores to a three-level risk assessment (HIGH, MODERATE, LOW). With 13 benchmarked methods, convergence thresholds are expressed proportionally: HIGH risk requires agreement from a supermajority (≥70%, i.e., ≥10*/*13) of applicable methods, MODERATE from a simple majority (≥50%, i.e., ≥7*/*13), and LOW when fewer than half agree. These proportional thresholds apply to the *active* method pool—methods that can produce a signal given the available data. In the synthetic benchmark, four learning-dynamics methods (Equalized Odds, GroupDRO, GCE, SSA) do not activate because they require training-time loss dynamics absent in pre-computed synthetic embeddings, reducing the active pool to 8 methods. Consequently, the HIGH convergence threshold of ≥7*/*12 raw votes reported in Table 11 corresponds to ≥7 of 8 active methods (87.5%), which satisfies the ≥70% supermajority criterion when applied to the active pool.

1. **Indicator Count:** HIGH when ≥⌈0.7|ℳ|⌉ methods detect shortcuts; MODERATE when ≥⌈0.5|ℳ|⌉; LOW otherwise (originally: HIGH ≥ 2, MODERATE = 1 for 4 methods).
2. **Majority Vote:** Each method casts a binary vote. HIGH when ≥ high_threshold fraction of methods vote (default 70%); configurable quorum.
3. **Weighted Risk:** Each method is weighted by its evidence strength:
  - Probe: weighted by distance above chance level.
  - Statistical: weighted by proportion of significant comparisons after correction.
  - HBAC: weighted by cluster confidence score.
  - Geometric: weighted by maximum effect size (capped at 1.0).
  - Frequency, Bias Direction PCA, SIS: weighted by normalized detection scores.
  - Demographic Parity, Equalized Odds, Intersectional: weighted by disparity magnitude.
  - GroupDRO: weighted by worst-group loss gap.
  - GCE: weighted by minority sample loss ratio.
  - SSA: weighted by spectral shift magnitude.
    The weighted average maps to risk levels via thresholds (default: HIGH ≥ 0.6, MODERATE ≥ 0.3).
4. **Multi-Attribute Intersection:** Runs detection per sensitive attribute and flags HIGH when ≥k attributes show independent risk (default *k* = 2). This captures intersectional shortcuts.
5. **Meta-Classifier:** A logistic regression (C=0.5, balanced class weights) trained on 800 synthetic scenarios across controlled difficulty levels. The model uses 26 features extracted from all detector outputs, including per-method success flags, risk indicator counts, and method-specific metrics (probe accuracy, statistical significance ratio, HBAC confidence, geometric effect sizes). Cross-validated accuracy: 98.1%. Decision thresholds: HIGH ≥ 0.978, MODERATE ≥ 0.202 (optimized on validation set). Falls back to a heuristic ensemble (0.6 × avg_risk + 0.4 × indicator signal) when the trained model is unavailable.

## Appendix C. Default Detection Thresholds

Table 8 documents the default thresholds used by each detection method to classify risk levels. These thresholds are empirically derived and configurable via the *ShortKit-ML* API.

**Table 8.** Default detection thresholds by method.

Method	HIGH threshold	MODERATE threshold
Probe accuracy	> 85%	> 70%
Statistical (sig. dims)	> 30% of dims	> 10% of dims
HBAC purity	> 80% + linear	> 65%
Geometric effect size	> 0.8 (Cohen’s d)	> 0.5
Frequency sensitivity	> 0.3 (norm. shift)	> 0.1
Bias Direction PCA gap	> 0.8 (proj. gap)	> 0.5
SIS (min. sufficient dims)	< 5% of dims	< 15% of dims
Equalized Odds gap	> 0.15	> 0.05
Demographic Parity gap	> 0.15	> 0.05
Intersectional disparity	> 0.20	> 0.10
GroupDRO worst-group gap	> 0.30	> 0.15
GCE minority loss ratio	> 2.0	> 1.5
SSA spectral shift	> 0.50	> 0.25

## Appendix D. Implementation and Reproducibility

*ShortKit-ML* is distributed as a pip-installable Python package. All benchmark experiments are configured through a JSON specification that defines the full parameter grid (effect sizes, sample sizes, embedding dimensions, imbalance ratios, random seeds, correction methods). The benchmark runner executes the complete grid and outputs structured JSON and CSV results for downstream analysis and figure generation.

The synthetic data generator produces reproducible embeddings with controlled shortcut characteristics via seeded random number generation. All statistical tests use the same correction pipeline (Bonferroni, Holm, FDR-BH, FDR-BY via statsmodels). Scripts for reproducing baseline comparisons against AIF360 and Fairlearn are included in the repository.

Source code, documentation, example notebooks, and pre-trained meta-classifier weights are publicly available at https://github.com/criticaldata/ShortKit-ML.

## Appendix E. Mitigation Method Details

1. **Shortcut Masking:** Zeroes, randomizes, or inpaints embedding dimensions identified as shortcuts by the detection pipeline. Operates at the data level and requires no retraining.
2. **Background Randomization:** For image-level pipelines, swaps foreground objects across different backgrounds to break spurious background–label correlations. Requires access to segmentation masks or foreground extraction.
3. **Adversarial Debiasing:** Adversarial training with a gradient reversal layer to remove group information from learned representations [17, 38]. The adversary attempts to predict A from E; the encoder is trained to maximize adversary loss while maintaining task performance.
4. **Explanation Regularization (RRR):** Penalizes model attention on detected shortcut regions using a Right for the Right Reasons regularization term added to the training loss.
5. **Last-Layer Retraining (DFR):** Retrains only the final classification layer on a group-balanced data subset [31]. This approach is computationally lightweight and effective when shortcuts are encoded primarily in the classification head rather than the feature extractor.
6. **Contrastive Debiasing:** Applies a contrastive loss that encourages alignment of representations across demographic groups [59], reducing the demographic separability of the embedding space while preserving task-relevant structure.

## Appendix F. Synthetic Benchmark Detail

Table 9 reports false positive rates on null data for all 12 benchmarked methods (intersectional excluded from synthetic evaluation). Table 10 summarizes sensitivity analysis. Table 11 compares convergence thresholds.

**Table 9.** False positive rates on null data (δ=0) across embedding dimensionalities (n=1000, 10 seeds) for all 12 benchmarked methods. Three methods (Bias Dir., SIS, Dem. Parity) have high individual FPR; HIGH-agreement convergence (≥7/12) suppresses FP to 0.000 across all dimensions.

Method	d=128	d=256	d=512
HBAC	0.000	0.000	0.000
Probe	0.000	0.000	0.000
Statistical	0.000	0.000	0.200
Geometric	0.000	1.000	1.000
Frequency	0.000	0.000	1.000
Bias Dir. (PCA)	1.000	1.000	1.000
SIS	1.000	1.000	1.000
Dem. Parity	1.000	1.000	1.000
Eq. Odds	0.000	0.000	0.000
GroupDRO	0.000	0.000	0.000
GCE	0.000	0.000	0.000
SSA	0.000	0.000	0.000
Conv. (≥7/12)	0.000	0.000	0.000

**Table 10.** Sensitivity analysis: detection rate at δ=0.8 across sample size, class imbalance, and dimensionality (multiple seeds, d=128 base) for all 12 benchmarked methods. Methods that never flag (Eq. Odds, GCE, Group-DRO, SSA) are learning-dynamics/fairness variants sensitive only to stronger or domain-specific signal.

Method	n=200	n=1000	n=5000	imb=0.5	imb=0.9	d=128	d=256	d=512
	*Sample Size*			*Imbalance*		*Dimensionality*		
HBAC	0.60	1.00	1.00	1.00	1.00	1.00	1.00	0.30
Probe	1.00	1.00	1.00	1.00	1.00	1.00	1.00	1.00
Statistical	1.00	1.00	1.00	1.00	1.00	1.00	1.00	1.00
Geometric	1.00	1.00	1.00	1.00	1.00	1.00	1.00	1.00
Frequency	1.00	1.00	1.00	1.00	1.00	1.00	1.00	1.00
Bias Dir. (PCA)	1.00	1.00	1.00	1.00	1.00	1.00	1.00	1.00
SIS	1.00	1.00	1.00	1.00	1.00	1.00	1.00	1.00
Dem. Parity	1.00	1.00	1.00	1.00	1.00	1.00	1.00	1.00
Eq. Odds	0.00	0.00	0.00	0.00	0.00	0.00	0.00	0.00
GroupDRO	0.00	0.00	0.00	0.00	0.00	0.00	0.00	0.00
GCE	0.00	0.00	0.00	0.00	0.00	0.00	0.00	0.00
SSA	0.00	0.00	0.00	0.00	0.00	0.00	0.00	0.00

**Table 11.** Convergence threshold comparison: accuracy (Acc), false positive rate (FP), and false negative rate (FN) across difficulty levels (n=1000,d=128, multiple seeds, 12 methods). Indicator Count has FP=1.0 at δ=0; HIGH agreement (≥7/12) achieves FP=0.0 everywhere and full detection at δ≥0.5.

Aggregation	Clean (δ=0)			Weak (δ=0.2)			Mod. (δ=0.5)			Strong (δ=0.8)		
	Acc	FP	FN	Acc	FP	FN	Acc	FP	FN	Acc	FP	FN
Indicator Count (≥1)	.00	1.0	.00	1.0	.00	.00	1.0	.00	.00	1.0	.00	.00
LOW (≥4/12)	1.0	.00	.00	.42	.00	.58	1.0	.00	.00	1.0	.00	.00
MODERATE (≥5/12)	1.0	.00	.00	.00	.00	1.0	1.0	.00	.00	1.0	.00	.00
HIGH (≥7/12)	1.0	.00	.00	.00	.00	1.0	1.0	.00	.00	1.0	.00	.00

### F.1. Harder Synthetic Variants.

To address potential reviewer concerns about synthetic realism, we benchmarked two harder variants (n=1000,d=128,k=5,10seeds):

- **Correlated (ρ=0.7)**: shortcut dimensions form a correlated block (compound symmetry), reducing per-dimension power.
- **Distributed**: effect spread uniformly across all d=128 dimensions at per-dim shift $\delta \sqrt{k/d}$, preserving total signal energy.

Table 12 shows detection rates. At δ=0.8 all methods remain robust. At δ=0.2, convergence drops to 0/10 for the correlated variant (geometric and HBAC lose sensitivity) and 8/10 for the distributed variant, confirming that multi-perspective agreement is critical under harder shortcut geometries.

## Appendix G. Statistical Detail for Case Studies

Table 13 provides statistical robustness analysis for the 3 shared backbones across both datasets, covering sex and race attributes (age excluded). Table 15 reports permutation-based significance tests.

### G.1. Convergence Threshold Calibration.

Table 16 shows that qualitative conclusions are stable across agreement thresholds ≥4 and ≥5 out of 13. The sensitive boundary is ≥6: MIMIC-CXR race and age drop to undetected there, while CheXpert conclusions hold through ≥7. This confirms results are not an artifact of a specific threshold choice. Note: GroupDRO and SSA fail on frozen embeddings, reducing the effective method count to 11 of 13.

**Table 12.** Detection rate (10 seeds) on harder synthetic variants (n=1000,d=128,k=5). **Bold** = convergence ≥2 achieved.

δ	Variant	Probe	Stat.	HBAC	Geom.	Conv.≥2
0.2	Standard	0.10	1.00	0.00	1.00	**1.00**
	Correlated (ρ=0.7)	0.00	1.00	0.00	0.00	0.00
	Distributed	0.00	0.80	0.00	1.00	**0.80**
0.5	Standard	1.00	1.00	0.60	1.00	**1.00**
	Correlated (ρ=0.7)	0.10	1.00	0.00	1.00	**1.00**
	Distributed	1.00	1.00	0.90	1.00	**1.00**
0.8	Standard	1.00	1.00	1.00	1.00	**1.00**
	Correlated (ρ=0.7)	1.00	1.00	1.00	1.00	**1.00**
	Distributed	1.00	1.00	1.00	1.00	**1.00**

**Table 13.** Statistical robustness analysis for the 3 shared backbones (RAD-DINO, ViT-B/16, MedSigLIP) across both datasets, for sex and race attributes (age excluded as the binarization threshold reduces Cohen’s d interpretability for ordered attributes). Probe F1 (macro) with 95% bootstrap CI (5-fold CV, 2,000 resamples). Cohen’s d: mean per-dimension standardized effect size (binary attributes only). FDR-BH Sig.: number of individual group comparisons with at least one significant dimension after Benjamini-Hochberg correction (α=0.05). Seed stability: mean ± std of probe F1 across 5 independent random seeds. Sex detection is highly robust (F1>0.80, seed std < 0.01) across all backbones; race probe F1 is lower (~0.40) but clustering, bias-direction, and fairness-based methods still detect race shortcuts (Table 4).

Dataset	Backbone	Attr.	N	Dim	Probe F1 (95% CI)	Cohen’s d	FDR-BH Sig.	Seed Stability
CheXpert	ResNet-50	Sex	2,000	2048	0.810 [0.797, 0.823]	0.087	1/1	0.811 ± 0.002
CheXpert	ResNet-50	Race	2,000	2048	0.403 [0.381, 0.425]	·	10/10	0.404 ± 0.002
CheXpert	DenseNet	Sex	2,000	1024	0.847 [0.829, 0.864]	0.136	1/1	0.845 ± 0.005
CheXpert	DenseNet	Race	2,000	1024	0.480 [0.458, 0.503]	·	10/10	0.475 ± 0.006
CheXpert	ViT-B/16	Sex	2,000	768	0.819 [0.809, 0.831]	0.107	1/1	0.823 ± 0.004
CheXpert	ViT-B/16	Race	2,000	768	0.455 [0.443, 0.467]	·	10/10	0.445 ± 0.011
MIMIC	RAD-DINO	Sex	1,491	768	0.990 [0.986, 0.994]	0.146	1/1	0.991 ± 0.001
MIMIC	RAD-DINO	Race	1,491	768	0.411 [0.386, 0.435]	·	10/10	0.412 ± 0.014
MIMIC	ViT-16	Sex	1,491	768	0.867 [0.861, 0.873]	0.137	1/1	0.866 ± 0.004
MIMIC	ViT-16	Race	1,491	768	0.390 [0.369, 0.408]	·	10/10	0.354 ± 0.025
MIMIC	MedSigLIP	Sex	1,491	1152	0.966 [0.958, 0.972]	0.302	1/1	0.966 ± 0.001
MIMIC	MedSigLIP	Race	1,491	1152	0.368 [0.333, 0.399]	·	10/10	0.373 ± 0.008

**Table 14.** Dataset demographics, geometric effect sizes, and probe accuracy with 95% bootstrap confidence intervals (5-fold CV, 2,000 bootstrap resamples, seed 42). Cohen’s d is the mean absolute per-dimension standardized difference between group centroids. FDR-BH Sig. reports embedding dimensions with significant group differences after Benjamini–Hochberg correction (α=0.05). Age is binarized as ≥60 vs <60. MIMIC-CXR race is binarized as WHITE vs non-WHITE.

Dataset	Attr.	N	Ng_0_/N_g1_	Dim	Cohen’s d	Probe Acc (95% CI)	FDR-BH Sig.
CheXpert (ResNet-50)	Race	2,000	850/1150	2048	0.070	0.558 [0.532, 0.583]	301/2048
CheXpert (ResNet-50)	Sex	2,000	1149/851	2048	0.087	0.793 [0.752, 0.822]	597/2048
CheXpert (ResNet-50)	Age	2,000	1039/961	2048	0.099	0.703 [0.673, 0.733]	770/2048
CheXpert (DenseNet)	Race	2,000	850/1150	1024	0.098	0.562 [0.544, 0.591]	407/1024
CheXpert (DenseNet)	Sex	2,000	1149/851	1024	0.136	0.759 [0.720, 0.797]	530/1024
CheXpert (DenseNet)	Age	2,000	1039/961	1024	0.152	0.665 [0.625, 0.705]	590/1024
CheXpert (ViT-B/16)	Race	2,000	850/1150	768	0.089	0.551 [0.526, 0.569]	264/768
CheXpert (ViT-B/16)	Sex	2,000	1149/851	768	0.107	0.790 [0.770, 0.812]	332/768
CheXpert (ViT-B/16)	Age	2,000	1039/961	768	0.131	0.659 [0.633, 0.685]	435/768
MIMIC (RAD-DINO)	Race	1,491	464/1027	768	0.081	0.742 [0.716, 0.770]	114/768
MIMIC (RAD-DINO)	Sex	1,491	767/724	768	0.146	0.991 [0.989, 0.993]	386/768
MIMIC (RAD-DINO)	Age	1,491	614/877	768	0.117	0.822 [0.799, 0.845]	283/768
MIMIC (ViT-16)	Race	1,491	464/1027	768	0.097	0.707 [0.682, 0.728]	200/768
MIMIC (ViT-16)	Sex	1,491	767/724	768	0.137	0.867 [0.847, 0.887]	395/768
MIMIC (ViT-16)	Age	1,491	614/877	768	0.184	0.748 [0.732, 0.766]	498/768
MIMIC (MedSigLIP)	Race	1,491	464/1027	1152	0.126	0.734 [0.719, 0.749]	479/1152
MIMIC (MedSigLIP)	Sex	1,491	767/724	1152	0.302	0.962 [0.954, 0.970]	869/1152
MIMIC (MedSigLIP)	Age	1,491	614/877	1152	0.309	0.824 [0.796, 0.845]	914/1152
CelebA	Male	10,000	5819/4181	2048	0.426	0.943 [0.941, 0.946]	1921/2048

**Table 15.** Probe permutation test ($n_{\text{perm}}=100$, seed 42). Observed probe accuracy versus null distribution from 100 label-shuffled permutations. p<0.01 indicates the embedding encodes group membership beyond chance. CheXpert rows were regenerated with per-backbone embeddings matching Table 13; all now show p<0.01, consistent with the strong probe F1 reported there.

Dataset	Attribute	Obs. Acc	Null ± Std	p
CheXpert (ResNet-50)	Race	0.545	0.514 ± 0.013	<0.01
CheXpert (ResNet-50)	Sex	0.786	0.514 ± 0.014	<0.01
CheXpert (ResNet-50)	Age	0.693	0.500 ± 0.015	<0.01
CheXpert (DenseNet)	Race	0.573	0.512 ± 0.012	<0.01
CheXpert (DenseNet)	Sex	0.745	0.512 ± 0.014	<0.01
CheXpert (DenseNet)	Age	0.647	0.499 ± 0.015	<0.01
CheXpert (ViT-B/16)	Race	0.544	0.510 ± 0.012	<0.01
CheXpert (ViT-B/16)	Sex	0.787	0.511 ± 0.013	<0.01
CheXpert (ViT-B/16)	Age	0.665	0.501 ± 0.014	<0.01
MIMIC (RAD-DINO)	Race	0.736	0.565 ± 0.017	<0.01
MIMIC (RAD-DINO)	Sex	0.990	0.500 ± 0.016	<0.01
MIMIC (RAD-DINO)	Age	0.818	0.517 ± 0.013	<0.01
MIMIC (ViT-16)	Race	0.695	0.577 ± 0.014	<0.01
MIMIC (ViT-16)	Sex	0.855	0.503 ± 0.016	<0.01
MIMIC (ViT-16)	Age	0.740	0.517 ± 0.016	<0.01
MIMIC (MedSigLIP)	Race	0.735	0.635 ± 0.010	<0.01
MIMIC (MedSigLIP)	Sex	0.960	0.500 ± 0.017	<0.01
MIMIC (MedSigLIP)	Age	0.810	0.536 ± 0.013	<0.01
CelebA	Male	0.920	0.520 ± 0.013	<0.01

**TABLE 16.** Convergence threshold calibration: fraction of backbone–attribute combinations classified as “detected” at each agreement cutoff. CheXpert: 21 combinations (7 backbones × 3 attr.); MIMIC-CXR: 12 combinations ( 4 backbones × 3 attr.).

Dataset	Attribute	Obs. n/13	≥4	≥5	≥6	≥7	≥8
CheXpert	Sex	6–8	1.00	1.00	1.00	1.00	0.71
CheXpert	Age	6–7	1.00	1.00	1.00	1.00	0.00
CheXpert	Race	6–7	1.00	1.00	1.00	1.00	0.00
MIMIC	Sex	5–6	1.00	1.00	0.25	0.00	0.00
MIMIC	Age	5	1.00	1.00	0.00	0.00	0.00
MIMIC	Race	5	1.00	1.00	0.00	0.00	0.00

## Appendix H. Per-Diagnosis Stratified Analysis

Table 17 reports shortcut detection results using all 13 methods when filtering CheXpert to patients with each specific diagnosis (attribute = sex). The top five diagnoses by prevalence are shown across all 7 backbones, with agreement ranging from 4/13 to 8/13, indicating that sex-related shortcut signals persist within diagnosis-specific subsets.

**Table 17.** Per-diagnosis shortcut detection on CheXpert (attribute = sex) using 13 methods across 7 backbones. Top 5 diagnoses by prevalence. Each cell: ✓ = method flagged shortcut, ・ = not flagged. Subsets are filtered to patients with the given diagnosis.

Diagnosis	Backbone	n	HBAC	Probe	Stat.	Geom.	Freq.	BiasDir	SIS	DP	EO	Inter.	Agree.
Support Devices	ResNet-50	933	✓	✓	·	·	✓	✓	✓	✓	✓	✓	8/13
	DenseNet-121	933	✓	✓	·	·	✓	✓	✓	✓	✓	✓	8/13
	ViT-B/16	933	✓	✓	·	·	✓	✓	✓	✓	✓	✓	8/13
	ViT-B/32	933	✓	✓	·	·	✓	✓	✓	✓	·	✓	7/13
	DINOv2	933	✓	✓	·	·	✓	✓	✓	✓	✓	✓	8/13
	RAD-DINO	933	✓	✓	·	·	✓	✓	✓	✓	·	✓	7/13
	MedSigLIP	933	✓	·	·	·	✓	✓	✓	✓	·	✓	6/13
Lung Opacity	ResNet-50	827	✓	✓	·	·	✓	✓	✓	✓	✓	✓	8/13
	DenseNet-121	827	✓	✓	·	·	✓	✓	✓	✓	✓	✓	8/13
	ViT-B/16	827	✓	✓	·	·	✓	✓	✓	✓	·	✓	7/13
	ViT-B/32	827	✓	✓	·	·	✓	✓	✓	✓	·	✓	7/13
	DINOv2	827	✓	✓	·	·	✓	✓	✓	✓	·	✓	7/13
	RAD-DINO	827	✓	✓	·	·	✓	✓	✓	✓	·	✓	7/13
	MedSigLIP	827	✓	·	·	·	✓	✓	✓	✓	·	✓	6/13
Pleural Effusion	ResNet-50	730	✓	✓	·	·	✓	✓	✓	✓	·	✓	7/13
	DenseNet-121	730	✓	✓	·	·	✓	✓	✓	✓	·	✓	7/13
	ViT-B/16	730	✓	✓	·	·	✓	✓	✓	✓	·	✓	7/13
	ViT-B/32	730	✓	✓	·	·	✓	✓	✓	✓	·	✓	7/13
	DINOv2	730	✓	✓	·	·	✓	✓	✓	✓	·	✓	7/13
	RAD-DINO	730	✓	✓	·	·	✓	✓	✓	✓	·	✓	7/13
	MedSigLIP	730	✓	·	·	·	✓	✓	✓	✓	·	✓	6/13
Edema	ResNet-50	366	✓	✓	·	·	✓	✓	✓	·	·	·	5/13
	DenseNet-121	366	✓	✓	·	·	✓	✓	✓	·	·	·	5/13
	ViT-B/16	366	✓	✓	·	·	✓	✓	✓	·	·	·	5/13
	ViT-B/32	366	✓	✓	·	·	✓	✓	✓	·	·	·	5/13
	DINOv2	366	✓	✓	·	·	✓	✓	✓	·	·	·	5/13
	RAD-DINO	366	✓	✓	·	·	✓	✓	✓	·	·	·	5/13
	MedSigLIP	366	✓	·	·	·	✓	✓	✓	·	·	·	4/13
Atelectasis	ResNet-50	266	✓	·	·	·	✓	✓	✓	✓	·	✓	6/13
	DenseNet-121	266	✓	✓	·	·	✓	✓	✓	✓	·	✓	7/13
	ViT-B/16	266	✓	✓	·	·	✓	✓	✓	✓	·	✓	7/13
	ViT-B/32	266	✓	✓	·	·	✓	✓	✓	✓	·	✓	7/13
	DINOv2	266	✓	✓	·	·	✓	✓	✓	✓	·	✓	7/13
	RAD-DINO	266	✓	✓	·	·	✓	✓	✓	✓	·	✓	7/13
	MedSigLIP	266	✓	·	·	·	✓	✓	✓	✓	·	✓	6/13

## Appendix I. GradCAM Attention Analysis on CelebA

To complement the embedding-space evidence, we apply GradCAM [47] on the CelebA Male-attribute task. A linear probe is trained on the 2,048-d ResNet-50 embeddings (training accuracy 94.7%) and attached to the full ResNet-50 backbone to form a differentiable end-to-end model. GradCAM is computed at layer4 for 16 sample images (8 Male, 8 Female), targeting the Male class.

We measure the *face-attention ratio* (FAR): the fraction of total GradCAM activation inside the annotated face bounding box. Male-predicted images yield FAR=0.48 on average, while Female-predicted images yield FAR=0.09—indicating that the model concentrates on facial structure when predicting Male and distributes attention more broadly (hair, background) for Female predictions. The overall mean FAR of 0.29 is consistent with the shortcut hypothesis: the model has learned to use gender-indicative facial features rather than disease-relevant regions, even on a non-medical dataset.
